# New insights from Norwegian and Swedish sports coaches' employment, practices, and beliefs during the first COVID-19 restriction period

**DOI:** 10.3389/fspor.2023.1277228

**Published:** 2023-10-27

**Authors:** Anna Cecilia Severin, Knut Skovereng, Glenn Björklund, Liv Hemmestad, Øyvind Sandbakk, Silvana Bucher Sandbakk

**Affiliations:** ^1^ Department of Neuromedicine and Movement Science, Centre for Elite Sports Research, Norwegian University of Science and Technology, Trondheim, Norway; ^2^ Department of Health Sciences, Swedish Winter Sports Research Centre, Mid Sweden University, Östersund, Sweden; ^3^ Department of Sport, Physical Education and Outdoor Studies, Faculty of Humanities, Sports and Educational Science, University of South-Eastern Norway, Bø in Telemark, Norway; ^4^ Department for Teacher Education, Norwegian University of Science and Technology, Trondheim, Norway

**Keywords:** lockdown, social distancing, training, Scandinavia, home confinement

## Abstract

**Introduction:**

This study (i) examined Norwegian and Swedish sports coaches' employment, practices, and beliefs during the first wave of the COVID-19 pandemic, (ii) compared these aspects between coaches in Norway and Sweden, two countries with clearly different movement restrictions strategies in this period.

**Methods:**

An online survey was distributed to coaches via email and social media. The survey was open between June and August 2020. In total, 348 coaches responded, 141 from Norway, and 207 from Sweden.

**Results:**

Among responders, 2% had lost their job due to the pandemic, 17% had been furloughed, 28% worked from home office, and 39% worked as usual. Norwegian coaches were more likely to work from home (48% vs. 15%, *p* < .001), while Swedish coaches were more likely to work as usual (60% vs. 9%, *p* < .001). Coaches in both countries communicated less frequently with their athletes (*p* < .001) and had less in-person communication (*p* < .001) compared to pre-Covid levels. Larger declines existed among Norwegian coaches regarding communication frequency (*p* < .001) and in-person communication (*p* < .001). Video calls and phone calls usage increased (*p* < .001 and *p* = .009 respectively). We recorded low levels of concern among coaches about the effects of the pandemic on their relationship with their athletes. There were considerable levels of concern about athletes’ maintaining their motivation to train (Norway: 43.3%, Sweden: 50.7%), and low levels of concern about the coaches’ relationships with their athletes (Norway: 14.1%, Sweden: 17.8%).

**Discussion:**

Overall, this study showed the imposed movement restrictions had several negative consequences for the employment and work practices of sports coaches in Norway and Sweden. However, it also highlighted that coaches were able to adapt their work practices to the constraints and were able to maintain relationships with their athletes. The consequences raised in this paper can act as a guide during possible future lockdowns.

## Introduction

Four months after the SARS-CoV-2 virus (COVID-19) was first identified, the World Health Organization declared COVID-19 a global pandemic. Governments around the world adopted restrictive measures to curb the spread of the disease, changing the day-to-day life for most people. While the nature and severity of these restrictions differed between countries, they often included closing schools, enforcing social distancing, and prohibiting public gatherings of more than a few people. Consequently, many industries struggled to cope and were forced to shut down or reduce their staff, leaving workers without a job or furloughed (1, 2). Reports from both the USA and mainland Europe have shown that the hospitality, leisure, retail, and entertainment sectors were among those hit hardest by unemployment and reduced work hours (3–5). The decreased employment was attributed to social distancing measures such as administrative closings, school closings, and confinement (5).

One of the many industries that were affected by the restrictions was the sporting industry. While Fana, Tolan et al. (4), indicated that sport can be included in the leisure sector, none of the reports detailed the effects of the pandemic on the sporting industry directly. However, the COVID-19 pandemic caused the most extensive disruption to sports practices since World War II (6), with world-wide cancelled competitions, halted seasons, and closed training facilities (7). Despite this, athletes still had to maintain their training, often on their own (8), in preparation for the staggered restart of competitive sport (9). A large number of reports highlight the many negative effects of the pandemic on athletes, such as increased risk of injury fur to insufficient training stimuli (e.g., 7, 8), poor nutrition, decreased motivation, and altered sleep patterns (10). Indeed, since the lifting of the restrictions, several research reports have shown higher injury rates amongst athletes than what was seen before the pandemic (e.g., 11–13). It is possible that cancelled seasons and competitions affected the employment of coaches, which, in turn contributed to these negative effects on the athletes.

In contrast to the many studies on athletes, the effects on the sports coaches are less examined and remains unclear (14). One recent study by Battaglia and Kerr (14) asked Canadian sports coaches how they perceived they were affected by the pandemic. The responses highlighted several concerning consequences, such as insufficient support services, lack of interactions with athletes, negative effects on coaches' mental health, and financial instability. On the other hand, the literature highlight that the pandemic provided coaches with an opportunity to further their own professional development and adapt their practice to new (online) formats (14, 15). The halted competitions, limited ability to meet and train in person, and a potentially increased risk of unemployment, meant that the coaches had to follow up their athletes as good as possible and quality assure the training process during the pandemic. They also had to appropriately plan the return to “normal” training without much experience or scientific knowledge to rely on (8). It remains unclear how these constraints affected how sports coaches perceived that their work tasks were accomplished and how their relationships with their athletes were affected.

Modern technology allows coaches to perform many aspects of their job digitally, such as communication, deliver training programs, and monitor training load, despite the restrictions (8). However, it is unclear how this technology has been adopted and how the responses to the pandemic have affected the working practices of sports coaches. Understanding how the pandemic affected sports coaches in terms of employment and coaching practices may help federations and governments to understand the consequences of the imposed restrictions on coaching and sport. Further, an insight into how sports coaches maintained the training of athletes during the COVID-19 pandemic would provide an understanding where the coaching profession stands due to the pandemic. This information can help guiding the development of strategies to create more robust organizations of sports in the future.

Norway and Sweden are two Scandinavian countries that are considered similar in many aspects, including political, social, economic, and cultural (16). While the Norwegian and Swedish sport organizations are based on similar foundations (17, 18), the governments’ strategies to tackle the first wave of the COVID-19 pandemic differed substantially. Norway closed schools and gyms, and cancelled all cultural and sporting events (16), with legal consequences imposed on those violating the restrictions (19). Sweden took a less restrictive approach where e.g., gyms and training facilities remained open (16), and the government relied more on the populations' own responsibility in controlling the spread of the virus through recommending social distancing measurements (20). While evaluating the success of these strategies in the short and long term is outside the scope of this paper. It is, however, likely that the different strategies affected the sporting industry, and coaches, differently during the first wave of the pandemic.

Therefore, the primary aim of this study was to examine how the first wave COVID-19 pandemic, during the spring and summer of 2020, affected the employment, work practices, and beliefs among sport coaches in Scandinavia, described from the coaches' perspective. It was hypothesized that the pandemic had negative effects on the coaches' employment and beliefs, and that they would adapt new work practices that included online communication and program prescription. The secondary aim was to compare how the different strategies employed by Norway and Sweden may be associated with different consequences for employment and work practices among sports coaches. Finally, aimed to evaluate learning points for sports coaches when facing similar situations.

## Materials and methods

### Overview

This study was conducted through an online open survey targeting sports coaches working in Norway and Sweden. The survey was created and reported according to The Checklist for Reporting Results of Internet E-Surveys (CHERRIES) (21), which is a well-established tool for conducting research from web-based questionnaires. Ethical approval for this study was obtained from the Norwegian Centre for Research Data in accordance with the institutional requirements and approval for data security and handling. The survey was created in Norwegian, Swedish, and English and was disseminated via Google Forms. The link to the survey was e-mailed to coaches via sports federations in Norway and Sweden, as well as shared via social media (e.g., Facebook and Twitter, Supplementary Material S1), and any active coach in either country could participate in the study. The first page of the survey contained information about the study and its purpose, clarified that participation was voluntary and that no incentives were offered in return for participation. This page had a mandatory tick-box to indicate participants' informed consent before proceeding to the questions. Participation was anonymous, coaches were not asked to provide their name or email address, and no identifying information, such as the IP address, was collected during the web-based data entry. It was consequently not possible to ensure that each coach only completed the survey once. Data was collected over a period of 51 days between the 29th of June 2020 and the 18th of August 2020.

### Data collection

The survey had 25 questions and was designed to assess employment, work practices, and beliefs among sports coaches in Scandinavia during the first wave of the COVID-19 pandemic. It was divided into three sections (pages), addressing participants' coaching background, communication with their athletes, and their practices during the pandemic (Supplementary Material S2). The Background section asked about: sex, sport, employment status (professional: 100% of salary from coaching, semi-professional: partial salary from coaching, or volunteer: no financial compensation), education, years of coaching experience, job situation during COVID-19 (worked as usual, worked as usual but from home-office, furloughed, lost job, or other), and whether they were affected by government-imposed movement restrictions at the time of answering the survey. The Communication section asked about the communication frequency with their athletes before and during the pandemic, and the methods used for communication before and during the pandemic. The Practices section asked about which tools they used to monitor training load and to deliver training programs during the pandemic and asked how they perceived that the situation had affected their athletes in terms of training quality, relationships with the coach and with other athletes, skill development, and motivation. To ensure that translated surveys were equivalent to the original version, which was written in English, the first draft was forwarded to other bilingual native researchers/colleagues for evaluation. The translated survey was sent back to the native researchers/colleagues who were required to recruit two participants to complete the survey and identify any concerns or difficulties, and this was adjusted accordingly.

### Statistical analysis

Statistical analysis was performed using Stata version 16.1 (StataCorp, TX, USA). Continuous and categorical variables are displayed as means (standard deviation, SD) and frequencies (percentage), respectively. For continuous variables, the Shapiro–Wilk test and standard visual inspection was used to examine the assumption of normality. Responses were converted to percentages to aid interpretation and comparison. An alpha level of ≤.05 was considered statistically significant.

Pairwise differences between countries were assessed with Pearson's chi-square tests of independence for proportions and independent samples *t*-tests for continuous variables. Changes in communication frequencies were assessed with Wilcoxon signed rank tests, both stratified within countries and in the whole sample. The Wilcoxon rank-sum test was used to assess between-country differences in change scores in communication frequency and level of concern for the athletes' maintenance of training during the pandemic. McNemar's test for dependent proportions was used to compare modes of communication before vs. during the pandemic. One-sample Wilcoxon signed-rank test were used to test the coaches' beliefs on the impacts of the COVID-19 pandemic on the athletes' training quality, relationships with the team, motivation to train, skill development, and relationship with athletes, using “neutral” on the Likert-scale questions as reference.

## Results

A total of 348 coaches responded to the survey, of which 141 (41%) were from Norway and 207 (59%) from Sweden (Table 1). We had coaches representing 43 sports, and 29 of these (67%) had less than 5 responding coaches. The largest sports were football *n* = 57, cross-country skiing *n* = 49, golf *n* = 40, shooting *n* = 29, and equestrian *n* = 28. Among all respondents, 25% were women, with a larger proportion among the Swedish coaches [X2(1, *n* = 347)] = 7.677, *p* = .006). Most of the responders (45%) worked professionally as coaches, meaning that all their income came from coaching, while 22% obtained part of their income from coaching (semi-professional) and 32% had a volunteer position, without financial compensation. There was no difference between countries in level of coaching position [*X*^2^(2, *n* = 345) = 3.274, *p* = .195]. A larger proportion of Norwegian coaches (58%) reported to have a university degree (i.e., Bachelor, Masters, or Doctorate), while 21% of Swedish coaches had an academic coaching education [*X*^2^(2, *n* = 348) = 48.153, *p* < .001]. No differences between countries were found for coaching experience, i.e., years of coaching (*t* = 1.268, *p* = .206).

**Table 1:** Sample characteristics of the responding coaches.

	Total	Norway	Sweden
Number of respondents	348	141 [41.0]	207 [59.0]
Gender			
Female	86 [24.7]	24 [17.0]	62 [30.0]
Male	261 [75.2]	117 [83.0]	144 [69.6]
Prefer not to say	1 [0.3]	0 [0.0]	1 [0.5]
Athletes			
Able-bodied athletes	278 [79.9]	111 [78.7]	167 [80.7]
Athletes with a disability	7 [2.0]	4 [2.8]	3 [1.5]
Both	63 [18.1]	26 [18.4]	37 [17.9]
Team or individual sport			
Individual	249 [71.6]	118 [83.7]	131 [63.3]
Team	85 [24.4]	17 [12.1]	68 [32.9]
Both/unclear	14 [4.0]	6 [4.3]	8 [3.9]
Number of athletes per coach			
Individual sports (*n* = 249)	18.8 ± 13.8	18.4 ± 13.9	19.1 ± 13.8
Team sports (*n* = 85)	11.6 ± 10.1	7.6 ± 10.5	12.6 ± 9.8
Coaching position			
Professional	156 [44.8]	71 [50.4]	85 [41.1]
Semi-professional	77 [22.1]	27 [19.2]	50 [24.2]
Volunteer	112 [32.2]	41 [29.1]	71 [34.3]
Another	3 [0.9]	2 [1.4]	1 [0.6]
Highest coaching qualification			
Ph.D.	3 [0.9]	2 [1.4]	1 [0.5]
M.Sc.	41 [11.8]	25 [17.9]	16 [7.7]
B.Sc.	81 [23.3]	54 [38.6]	27 [13.0]
Official certificate^a^	152 [43.8]	34 [24.3]	118 [57.0]
Other certificate^b^	40 [11.5]	16 [11.4]	24 [11.6]
Other/past athlete	2 [0.6]	1 [0.7]	1 [0.5]
None	28 [8.1]	8 [5.7]	20 [9.7]
Years of coaching	13.9 ± 10.8	13.0 ± 10.1	14.5 ± 11.2
Indoor or outdoor sport			
Indoor	82 [23.6]	52 [36.9]	30 [14.5]
Outdoor	208 [59.8]	76 [53.9]	132 [63.8]
Either	57 [16.4]	12 [8.5]	45 [21.7]
Other	1 [0.3]	1 [0.7]	0 [0.0]
Currently under restricted movement			
Yes	152 [43.7]	58 [41.1]	94 [45.4]
No	143 [41.1]	70 [49.7]	73 [35.3]
Never had restricted movement	53 [15.2]	13 [9.2]	40 [19.3]

Data is shown as: N [percent within total sample or country] or mean ± SD.

^a^ Official certificates refer to certification through an official organization (e.g., FIFA, FIS).

^b^ Other certificates refer to certification through other coaching courses.

At the time of the survey, 44% were under movement restrictions, while 42% were no longer under restriction (Table 1). There was no difference between countries in terms of how many coaches still experienced limited mobility at the time of participating in this study [45% vs. 41% for Sweden and Norway, respectively; *X*^2^(1, *n* = 348) = 0.623, *p* = .430]. Fifty-three (15%) coaches reported to not have had limited mobility at all during the pandemic, with most of these coaching in Sweden [19% vs. 9%, for Sweden and Norway respectively; *X*^2^(1, *n* = 348) = 12.003, *p* = .001].

**Figure 1:**
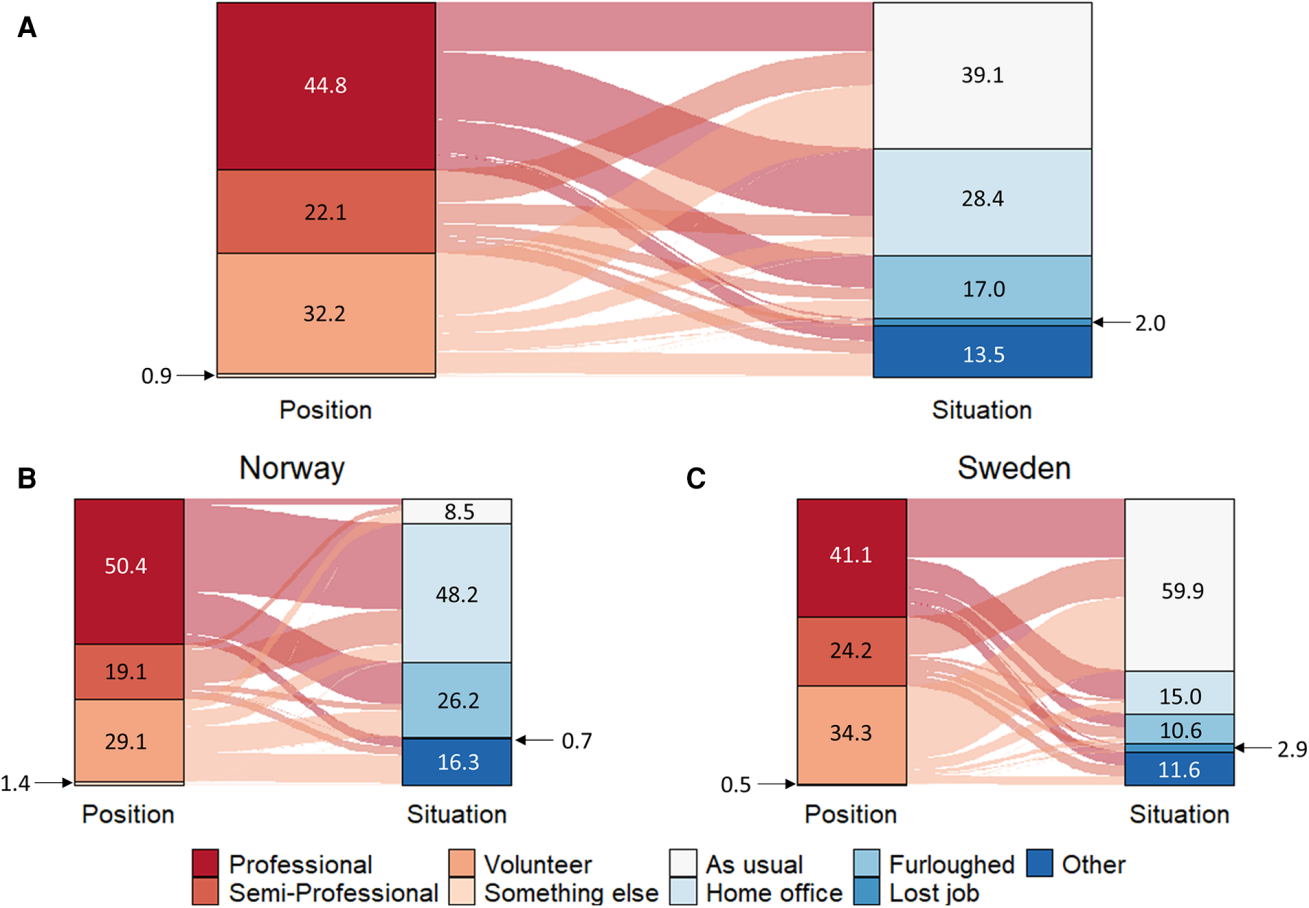
Alluvial diagram showing how the first wave of the COVID-19 pandemic affected the employment of (**A**) all coaches, (**B**) Norwegian coaches, and (**C**) Swedish coaches. Numbers represent % of responders. Situation “Other” includes Increased workload (*n* = 6, all Swedish), Decreased workload (*n* = 10, Norwegian = 4 and Swedish = 6), and No training (*n* = 12, all Norwegian). Number of responses per category is presented in Supplementary Material S3—Table 1.

Figure 1 shows the type of position (“Professional”, “Semi-Professional”, “Volunteer”, and “Something else”) and employment situation (“As usual”, “Home office”, “Furloughed”, “Lost job”, and “Other”) amongst the responding coaches.

Among all 348 coaches, 39% reported that their work continued as usual despite the COVID-19 pandemic, while 29% worked from home, 17% were furloughed, 2% lost their coaching jobs (Figure 1). Forty-seven (14%) coaches reported that their job position was affected in “other” ways than those listed in the survey (Figure 1), and the most frequent answers among those included “*increased workload*” (*n* = 6), “*decreased workload*” (*n* = 10) and “*no organized training*” (*n* = 12). A between-country comparison revealed that a significantly higher proportion of Swedish coaches reported that their job position remained unaffected by the pandemic compared to Norwegian coaches [56% vs. 9%, *X*^2^(1, *n* = 348) = 93.046, *p* < .001]. Additionally, Swedish coaches were less likely to be furloughed [11% vs. 26%, respectively; *X*^2^(1, *n* = 348) = 14.521, *p* < .001] or to work from home [15% vs. 48%, respectively; *X*^2^(1, *n* = 348) = 45.556, *p* < .001] compared to their Norwegian colleagues.

**Figure 2:**
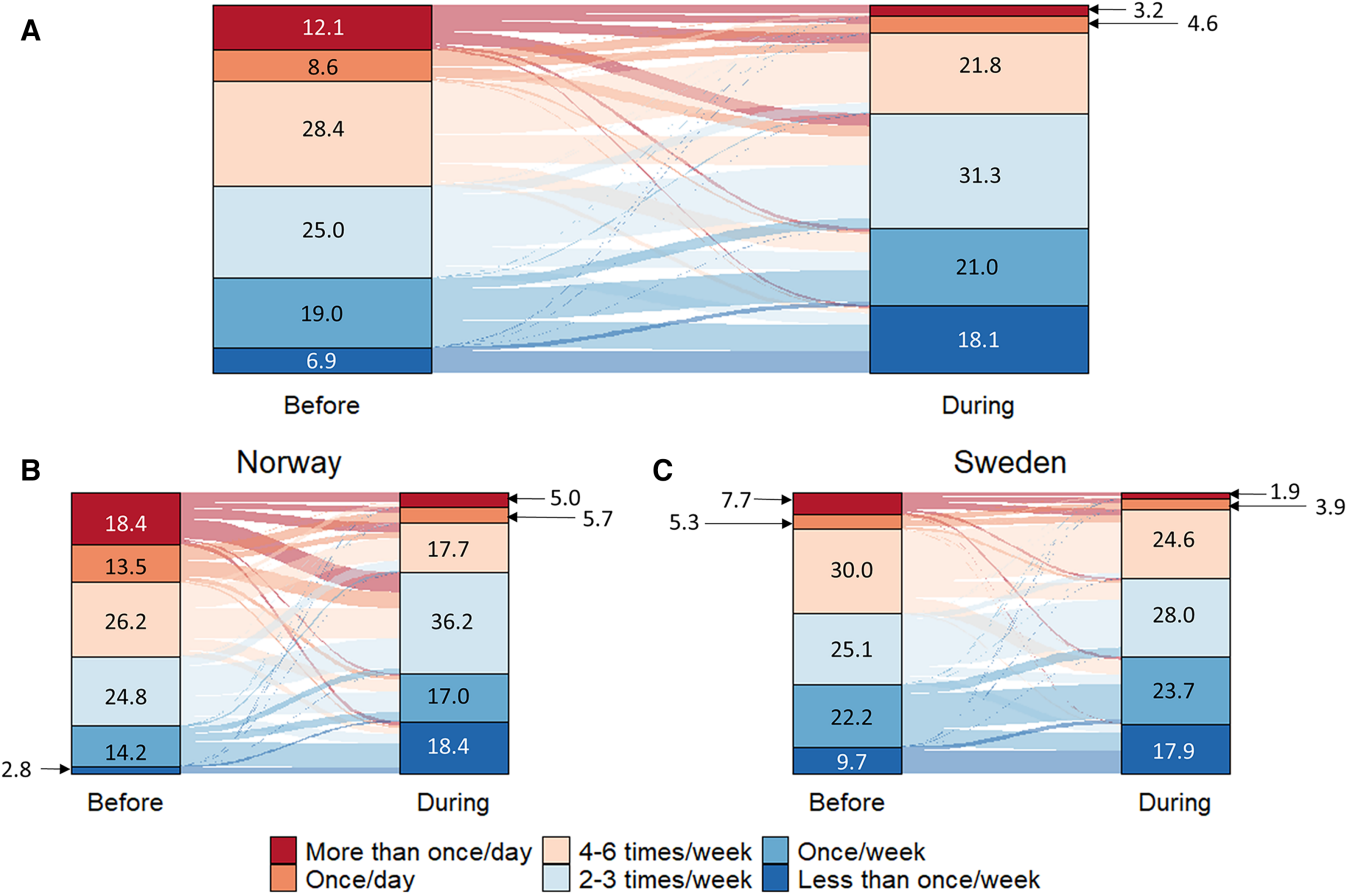
Alluvial diagrams of how the communication frequency changed due to the first wave of the 2020 COVID-19 pandemic for all responding coaches (**A**), Norwegian coaches (**B**) and Swedish coaches (**C**) numbers represent % of responders. Number of responses per category is presented in Supplementary Material S3—Table 1.

Figure 2 shows how often the coaches communicated with their athletes before and during the pandemic. The coaches communicated less frequently during the COVID-19 pandemic than before (*z* = −9.784, *p* < 0.001, within Norway: *z* = −7.533, *p* < .001; within Sweden: *z* = −6.161, *p* < .001) (Figure 2). However, the decline in communication frequency was significantly larger among Norwegian coaches compared to their Swedish colleagues (*z* = −4.590, *p* < .001).

There was a significant reduction in the amount of “in-person” communication among all coaches [89% before to 51% during the pandemic; McNemar's *X*^2^(1, *n* = 348) = 126.45, *p* < .001] (Table 2). This observed decline differed significantly by country (*z* = −6.304, *p* < .001), as 68% (83 of 123) of Norwegian coaches and 28% (53 of 188) of Swedish coaches completely ceased to communicate with their athletes in-person during the pandemic. The use of video calls [18% to 44%; McNemar's *X*^2^(1, *n* = 348) = 87.17, *p* < .001] and phone [57% to 61%; McNemar's *X*^2^(1, *n* = 348) = 6.74, *p* = .009] increased significantly among the responders, while no significant changes were observed in communication by email [53% to 56%; McNemar's *X*^2^(1, *n* = 348) = 2.78, *p* = .096] and text messages/social media [81% to 83%; McNemar's *X*^2^(1, *n* = 348) = 0.76, *p* = .384]. No significant differences were found between countries in changes of use of email (*z* = −0.658, *p* = .511), phone (*z* = 1.552, *p* = .121) or text messages/social media, except for the use of video calls, which increased more in Norway (*n* = 54 started) compared to Sweden (*n* = 39 started; *z* = 3.933, *p* < .001).

**Table 2:** Implications of the COVID-19 pandemic on communication methods and work practices.

	Total	Norway	Sweden
Communication methods before the pandemic			
E-mail	183 [52.6]	67 [47.5]	116 [56.0]
Video Call (e.g., Skype, Zoom, etc.)	62 [17.8]	33 [23.4]	29 [14.0]
Phone	197 [56.6]	83 [58.9]	114 [55.1]
Text messages and/or social media	281 [80.7]	115 [81.6]	166 [80.2]
In-person	311 [89.4]	123 [87.2]	188 [90.8]
Other	1 [0.3]	0 [0.0]	1 [0.5]
Communication methods during the pandemic			
E-mail	193 [55.5]	69 [48.9]	124 [59.9]
Video Call (e.g., Skype, Zoom, etc.)	153 [44.0]	86 [61.0]	67 [32.4]
Phone	213 [61.2]	94 [66.7]	119 [57.5]
Text messages and/or social media	289 [83.0]	118 [83.7]	168 [81.2]
In-person	177 [50.9]	40 [28.4]	137 [66.2]
Other	6 [1.7]	5 [3.5]	1 [0.5]
* No communication*	*4*	*4*	*0*
Person prescribing training programs during the pandemic			
Athlete writes their own programs	50 [14.4]	22 [15.6]	28 [13.5]
Coach	91 [26.2]	26 [18.4]	65 [31.4]
Collaboration between athlete and coach	176 [50.6]	80 [56.7]	96 [46.4]
Other	31 [8.9]	13 [9.2]	18 [8.7]
* No program*	*17*	*7*	*10*
* Other coach*	*9*	*5*	*4*
Method of delivering training plans and inspiration during the pandemic			
E-mail with written instructions	137 [39.4]	65 [46.1]	72 [34.8]
Links to pre-existing online videos	107 [30.7]	60 [42.6]	47 [22.7]
Online collaboration tools (e.g., Google docs, Teams, Slack, etc.)	126 [36.2]	68 [48.2]	58 [28.0]
Links to own online videos	66 [19.0]	26 [18.4]	40 [19.3]
None	86 [24.7]	17 [12.1]	69 [33.3]
Other	38 [10.7]	10 [7.1]	28 [13.5]
* In-person*	*20*	*2*	*18*
* Social media*	*7*	*5*	*2*
* Phone*	*3*	*1*	*2*
Method for monitoring athlete training during the pandemic			
Online diary	150 [43.1]	88 [62.4]	62 [30.0]
Smart technology (e.g., GPS, apps etc.)	42 [12.1]	23 [16.3]	19 [9.2]
Email/phone reports from athletes	189 [54.3]	82 [58.2]	107 [51.7]
Other	85 [24.4]	22 [15.6]	63 [30.4]
* In-person*	*44*	*3*	*31*
* Social media*	*12*	*9*	*3*
* No monitoring*	*39*	*11*	*28*
Most important sparring partner during the pandemic			
Athlete	157 [45.1]	64 [45.4]	93 [44.9]
Partner/spouse/family (not coach)	146 [42.0]	68 [48.2]	78 [37.7]
Friend (not coach)	49 [14.1]	20 [14.2]	29 [14.0]
Sports federation	68 [19.5]	22 [15.6]	29 [14.0]
Other coach	194 [55.7]	64 [45.4]	130 [62.8]
No one	46 [13.2]	22 [15.6]	24 [11.6]
Other	1 [0.3]	1 [0.7]	0 [0.0]
Level of concerned that the athletes have failed to maintain training during the pandemic			
Not at all concerned	127 [36.5]	41 [29.1]	86 [51.6]
Slightly concerned	88 [25.3]	39 [27.7]	49 [23.7]
Somewhat concerned	52 [14.9]	22 [15.6]	30 [14.5]
Moderately concerned	59 [17.0]	27 [19.2]	32 [15.5]
Very concerned	22 [6.3]	12 [8.5]	10 [4.8]

Data is shown as: N [percent within total sample or country]. the questions regarding methods for communication, delivering training plans, and athlete monitoring, along with the question regarding the most important sparring partner allowed multiple responses from each coach, so the number of responses does not add up to the number of coaches who responded to the survey.

In 51% of cases, the training programs were designed in collaboration between athletes and coaches, with no significant difference between countries [*X*^2^(1, *n* = 348) = 3.602, *p* = .058]. Cases where the coach alone prescribed training plans summed up to 26% and differed by country [31% vs. 18% for Sweden and Norway, respectively; *X*^2^(1, *n* = 348) = 7.296, *p* = .007]. Overall, training plans and inspirational content were most frequently delivered by e-mail (39%), online collaboration tools (36%) and via links to online videos (31%) during the pandemic. However, Swedish coaches were less likely to use these methods (*X*^2^[1, *n* = 348] = 4.500, *p* = .034; online collaboration tools: *X*^2^[1, *n* = 348] = 14.828, *p* < .001; and online videos: *X*^2^[1, *n* = 348] = 15.517, *p* < .001). Instead, 33% of Swedish coaches reported that they did not deliver any training plans and inspirational content to their athletes during the pandemic, which differed significantly compared to the Norwegian coaches [12%, *X*^2^(1, *n* = 348) = 20.407, *p* < .0.001]. Conversely, 18 Swedish coaches (9%) indicated in free-text answers that they deliver training plans and inspiration in-person, while only 2 (1%) Norwegian coaches reported in-person delivery.

The most frequently reported means of athlete monitoring were email/phone (54%) and online diaries (43%). While no significant differences existed between countries in the use of email/phone [*X*^2^(1, *n* = 348) = 1.413, *p* = .235], more Norwegian coaches used training diaries for athlete monitoring [62% vs. 30% for Norway and Sweden, respectively; *X*^2^(1, *n* = 348) = 36.033, *p* < .001]. Interestingly, the most frequent response with regards to monitoring training load amongst the responders was “none at all” (45%), with significantly higher proportion amongst the Swedish coaches (54% vs. 33% for Swedish and Norwegian coaches, respectively; [*X*^2^(1, *n* = 348) = 14.936, *p* < .001]. Among the 42% of coaches who used training diaries to monitor training load, a higher proportion was Norwegian (57%) compared to Swedish [31%; *X*^2^(1, *n* = 348) = 24.285, *p* < .001].

The most frequently reported persons for providing professional support and discussion for the coaches during the pandemic (henceforth referred to as “sparring partners”) were other coaches (56%), the athletes (45%) and partners/families (42%) (Table 2). Swedish coaches used other coaches as for support more frequently than Norwegian [*X*^2^(1, *n* = 348) = 10.307, *p* = .001], but no significant differences between countries was found for athletes [*X*^2^(1, *n* = 348) = 0.007, *p* = .932] and partner/family [*X*^2^(1, *n* = 348) = 3.830, *p* = .050]. In addition, Norwegian coaches reported a higher level of concern for how the pandemic has affected their athletes' training (*z* = 2.356, *p* = .019).

**Figure 3:**
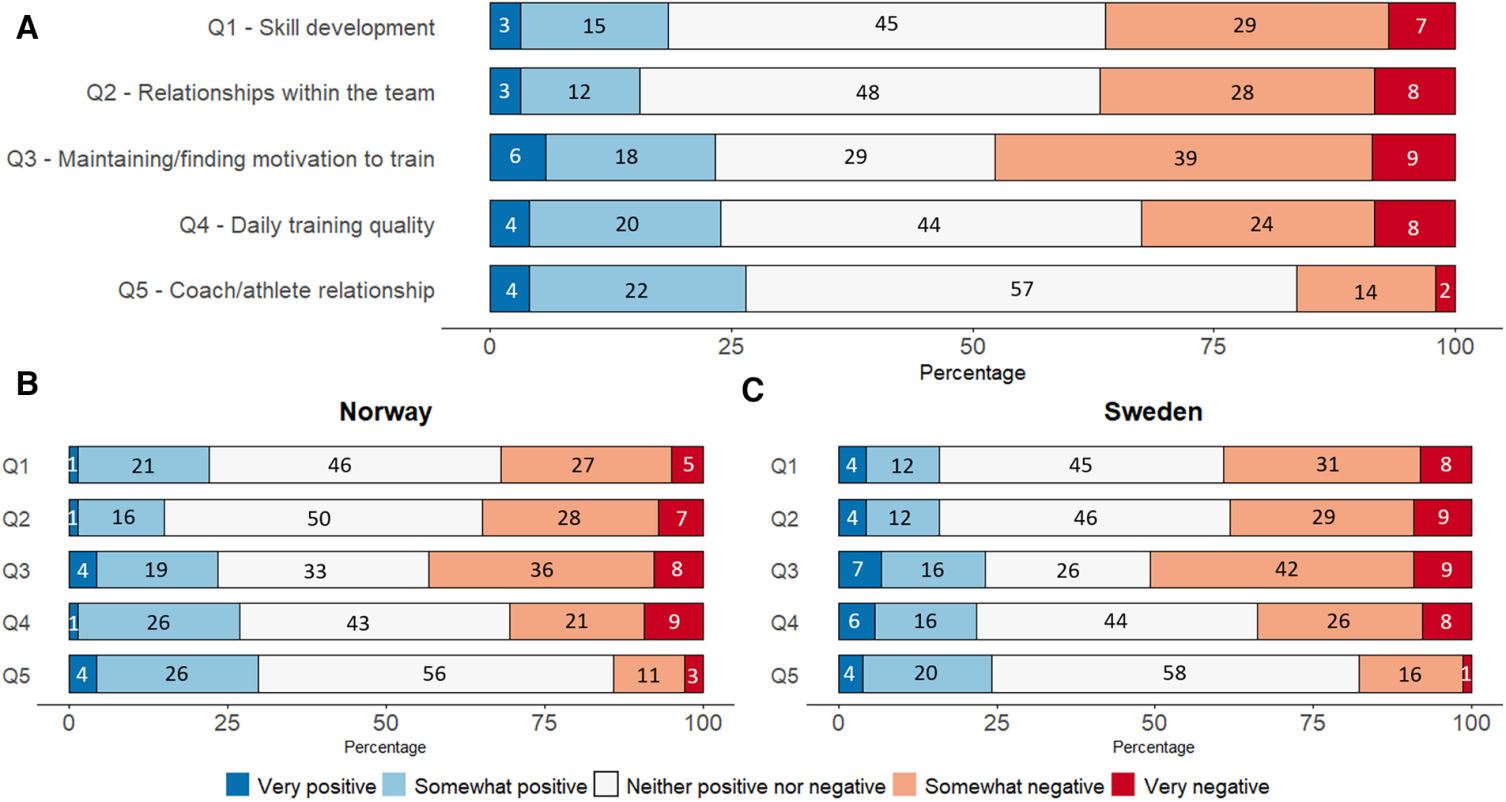
Illustration of how the coaches perceive the COVID-19 pandemic has affected their athletes. (**A**): all coaches, (**B**): Norwegian coaches, (**C**): Swedish coaches. Numbers represent % of responders for each category. Number of responses per category is presented in Supplementary Material S3—Table 2.

Figure 3 shows the frequency of responses on the Likert-scale questions, ranging from “very positive” through to “very negative”. One-sample Wilcoxon signed rank tests revealed a significantly negative perceived impact of the pandemic on the athletes' daily training quality (*z* = −2.348, *p* = .019), relationships with the team (*z* = −5.472, *p* < .001) and motivation to train (*z* = −5.036, *p* < .001) (Figure 3). No significant difference existed between countries for impact on training quality (*z* = 0.675, *p* = .499), relationships with the team (*z* = 0.388, *p* = .698) and motivation to train (*z* = 0.837, *p* = .383). Although the largest proportion of coaches reported that the pandemic neither positively or negatively affected the athletes' skill development (45%) or their own relationship with their athletes (57%), statistical analyses revealed that coaches perceived a significantly negative impact on skill development (*z* = −4.494, *p* < .001) and a positive impact on their relationships with their athletes (*z* = 2.900, *p* = .004), with no significant differences between countries (skill development: *z* = 1.639, *p* = .101; relationships: *z* = 1.241, *p* = .215).

## Discussion

This study assessed how the first wave (spring and summer of 2020) of the COVID-19 pandemic affected sports coaches in Norway and Sweden in terms of their employment situation, work practices and beliefs. Our first hypothesis was that the pandemic had negative effects on all three areas, which was supported by the data. The analysis showed that 61% of the sports coaches reported a change in their work situation and that almost half of the responders communicated less frequently with their athletes. The data also showed that the more liberal approach in Sweden allowed most coaches to continue to work “as usual” and to meet with their athletes in-person, while most Norwegian the majority of coaches worked “from home office” and used video calls to communicate with their athletes.

### Effects on employment

More than two thirds of the 348 participating coaches reported that they worked either “from their home office” or “as usual” during the time of the survey. Considering the reported high levels of unemployment and loss of work hours in the leisure and entertainment sectors (3, 4) (where sport arguably can be included), it is encouraging that most of our coaches continued to work during this time. While the unemployment levels in both Norway and Sweden increased during the first wave of the COVID-19 pandemic (16), only 2% of the coaches reported that they lost their employment, (Figure 1). However, close to one fifth (17%) of the coaches were furloughed, which testifies to the pandemic indeed having negative effects on the work hours in the profession. Considering that both Norway and Sweden cancelled sporting competitions and banned gatherings of more than a few people, it is not surprising that some coaches were left without work. Importantly, half of the coaches who were furloughed or lost their jobs worked professionally and thus relied fully on coaching for their income.

Coaches in Norway were at a higher risk of being furloughed than their Swedish colleagues (26.2% vs. 10.6%), which likely reflected Norway's stricter strategy (16). Almost 30% of Norwegian coaches reported that they were not working during the period referred to in the survey, while the equivalent number among Swedish coaches was 13.5%. Keeping coaches employed and working should be a priority since it may prevent many of the negative health effects that has been reported to originate from unemployment, such as boredom, and lack of social support, increased levels of stress, anxiety, and depression (16, 22, 23). It is therefore likely that a more liberal strategy would be beneficial for the well-being of coaches, especially during prolonged restrictions.

### Effects on work practices

#### Communication

In both nations, sport events were cancelled, and training was restricted during the first wave of the COVID-19 pandemic. However, outdoor physical activity and training remained unrestricted, thereby allowing athletes and coaches to maintain the training to some extent. Still, our analysis shows that coaches communicated less frequently with their athletes during the pandemic (48% of all coaches, Figure 2). Although this decrease was larger among Norwegian coaches (62% vs. 38% in Sweden), the considerable drop in communication frequency is concerning. Both elite and recreational athletes were shown to have reduced training load during the first wave of the pandemic (10, 24, 25), which together with the reduced communication frequency between athletes and coaches likely has contributed to the elevated injury rates reported in the seasons following the pandemic (11–13).

The methods of communication also shifted during the pandemic, with fewer coaches meeting their athletes “in-person” (before: 89%, during: 61%) and an increase in the use of video calls (such as Zoom and Skype, before: 18%, during: 44%) (Table 2). This trend has also been observed among Brazilian and Canadian coaches (14, 26), and can be seen across several professions, with more digital meetings and less physical contact. It is possible that the increased communication through video calls, in part, can compensate for the lack of meeting physically, since they allow for both verbal and nonverbal communication (27). Modern technology has certainly made it easier for coaches and athletes to communicate digitally, and a shift from text messages to video calls may have occurred even without the pandemic. The high proportion of responders that used electronic systems to deliver training plans and inspirational material to their athletes, and online tools for monitoring training loads, further shows that coaches generally transitioned well to digital solutions (Table 2). The benefits of an accelerated reliance on digital communication may extend even further: It has for example been suggested that coaches are increasingly in contact with both other practitioners and researchers (15), which may have large benefits for networking and the dissemination of new knowledge.

Further, Fana, Tolan et al. (4), suggested that jobs where employees were able to work digitally were likely to be less affected by the restrictions and closures compared to other sectors that experienced devastating effects. The authors specifically highlighted education and professional services as examples of jobs with the potential to work digitally and it appears that sports coaches were able to, at least partially, benefit from of this option. Digital communication is common in modern society, and the ease of contacting a coach or athlete likely enhances the relationship. Therefore, it is possible that the transition to increased reliance on modern technology would have occurred regardless of the pandemic and was merely accelerated by it.

The scientific literature on the quality of “online” vs. “in person” communication is currently biased to education and professional development (28, 29), with limited empirical analyses of the effects on coaching. While this literature highlights both positive and negative aspects of “online” delivery, it is not clear if these apply to sports coaches since they often have limited training in blended learning methods. Although it is possible that the quality may decline with prolonged digital communication, it is important to recognize that reduced communication frequency does not necessarily reflect a reduced communication quality. Regardless, this new environment where coaching has been moved to digital platforms to a larger extent may place larger emphasis of sports coaches receiving proper training in conducting online sessions and employing blended learning approaches into their practice.

#### Practices

More than of half of the coaches continued to collaborate with their athletes in developing the training programs, including communication, feedback, and follow-ups, during the pandemic (Table 2). It is possible that the coaches who maintained the communication with their athletes, also managed to maintain a positive coach-athlete relationship and could therefore be less concerned about the effects of the pandemic on performance-development. It is also likely that such an approach is beneficial for both the athletes' and coaches' mental wellbeing and may facilitate the transition back to pre-Covid training. This study did not assess the mental well-being of the coaches, however, Battaglia and Kerr (14) reported an increase in mental health challenges among the Canadian coaches, including perceived stress, depression, and isolation. This highlights the importance of establishing support systems for coaches so that they can seek help when needed.

The large proportion of coaches who reported that their athletes were among the most important sparring partners during the pandemic (Table 2) further highlights the important relationships between coaches and athletes and the lack of professional support systems. The support from their athletes was comparable to that from colleagues and family members and indicates a high level of trust between the coaches and their athletes. However, it is concerning that >10% of the coaches reported an absence of sparring partners during the pandemic. A lack of professional support may have a negative effect on their wellbeing and motivation to continue in the coaching role (30). In fact, less than 20% of coaches reported that they could rely on support from their federations. This issue was also highlighted by the Canadian coaches, who reported that the available support services during the pandemic were insufficient (14). Paoli and Musumeci (7) called for sporting federations worldwide to take a stand for athlete health during the pandemic but did not mention coaches and other support staff. While we support this call, also urge sporting federations and governing bodies to reach out to coaching staff and offer personal and professional support.

#### Beliefs

Many coaches in this study (48%), reported that they perceived the pandemic to have a “*negative*” or “*somewhat negative*” effect on the athletes' motivation to train (Figure 3). This was expected since the pandemic has been reported to negatively affecting athletes' motivation (10, 26, 31). However, motivation is complex and individual (30), so while research is showing a general negative effect of the pandemic, the effects will likely differ between individual athletes. Regardless, it is indisputable that the coach has an important role in developing and maintaining motivation in their athletes (32). The relationship between the coach and athlete has been described as “*one of the most important influences on athletes’ motivation and subsequent performance*” (30). It was somewhat unexpected that few coaches reported that the pandemic has had a “*negative*” (2%) or “*somewhat negative*” (14%) effect on their relationship with their athletes (Figure 3). Conversely, the Canadian coaches reported that the response to the pandemic had negative consequences on their personal connections with their athletes and that the digital solutions failed to compensate for the lack of personal interaction (14). However, based on our data, it seems coaches in Norway and Sweden were successful in maintaining a satisfactory relationships with athletes during the first wave of the pandemic and corresponding lockdowns.

It is encouraging that, the data showed relatively low levels of negative perceptions and concerns among coaches in how the pandemic affected their athletes (Table 2, Figure 3). This was in contrast to another study where coaches expressed concerns about the effects of the pandemic on athletic performance (26). It was unsurprising that more Swedish coaches were “*not at all concerned*” compared to Norwegian coaches, since more training facilities remained open in Sweden. Perhaps the more liberal strategy adopted in Sweden provided athletes with better opportunities to maintain their own training and thus caused lower concerns amongst the coaches.

### Limitations

This study is based on data collected during the first wave of the pandemic (spring and summer 2020) and it is likely that the prolonged time with updated and revised restrictions further affected the coaches beyond what is discussed here. A follow up study would provide valuable insights into the prolonged effects of the pandemic on sports coaches and offer an insight into whether current practices have changed as a consequence. In addition, using online surveys has some inherent risks, for example, the quality of self-reported data, and participants misunderstanding a question. Efforts were made to ensure quality by following specified suggestions (21), however, the anonymity of the questionnaire prevented us from contacting individual coaches and asking for clarification or elaboration. Finally, it is not known whether the adaptations made in response to the pandemic were temporary or resulted in a new standard, and whether, or to what extent, coaches and athletes considered them to be successful. Further investigations should assess the effects of these new practices and examine the extent to which they persist after the pandemic, as well as whether coaches have reverted to their pre-pandemic practices and communications methods.

## Conclusions

In this study, we show that the imposed movement restrictions had several negative consequences on the Norwegian and Swedish coaches in terms of lost work, less frequent communication with their athletes, a lack of support from their federations, and a concern about their athletes' ability to maintain their training. These consequences are concerning and it possible that they now contribute to the increased injury rates seen in post-pandemic sports. However, we also highlight some positive outcomes, such as the adaptability of coaches to work around the restrictions by relying on modern technology, and that they felt secure in their relationships with their athletes. Finally, we note some suggestions for measures that may help protect coaches, and similar professions, in case of future situations of a similar nature. These include establishing professional support systems, aiming to maintain employment to prevent negative side effects of unemployment, and developing strategies for conducting their work digitally. We further show that the more liberal approach in Sweden allowed more coaches to work as usual, keep communicating with their athletes in-person, and maintain work practices that were more similar to those used before the COVID-19 pandemic, which may reduce negative long-term consequences. These new insights into the effects of movement restrictions on Norwegian and Swedish sport coaches can help provide guidance during possible future lockdowns.

## Data Availability

The original contributions presented in the study are included in the article/**Supplementary Material**, further inquiries can be directed to the corresponding author.
