# Supplementary material for: New insights from Norwegian and Swedish sports coaches' employment, practices, and beliefs during the first COVID-19 restriction period
Source: Front Sports Act Living. 2023 Oct 27;5:1277228. doi: 10.3389/fspor.2023.1277228 (PMC10641774; doi:10.3389/fspor.2023.1277228)
Supplement: Supplementary file 2 [file Datasheet2.pdf]

Supporting information

Supplementary Material S1. Full questionnaire used for this study.

1. Your current place or residence (where you are or have been coaching during the Covid-19 pandemic). Please specify city and country (e.g., London, England)

____

2. What is your sex? (please chose one)

- ( ) Female
- ( ) Male
- ( ) Prefer not to say
- ( ) Other: ______________________________

3. What is your Sport? (the primary sport(s) you coach in) (please list all)

____

4. Do you coach athletes who are able-bodied or disabled? (please select one option)

- ( ) Able-bodied
- ( ) Disabled
- ( ) Both

5. Are you? (please select one option)

- ( ) Volunteer (not paid for coaching)
- ( ) Semi-Professional(income fromboth sportandanother activity)
- ( ) Professional (full income from your sport)
- ( ) Other: ______________________________

6. How was your job position (as a coach) affected by the Covid-19 pandemic? (please select one option)

- ( ) I lost my job (i.e., my position was terminated)
- ( ) I was furloughed (i.e., put on temporary suspension)
- ( ) I kept working as usual but from my home office
- ( ) I worked as usual (not from home office)
- ( ) Other: ______________________

7. How many years have you been coaching (regularly for) your sport? (please enter number)

____

8. How many individual athletes (not including team sport athletes) do you coach? (if none, type 0)

____

9. How many teams of athletes (not including athletes competing individually) do you coach? (if none, type 0)

____

10. What is/are your coaching qualification/s?

- [ ] Certificate/individual courses from non-official bodies
- [ ] Certificate/License from official body (FIFA, UEFA, IAAF, etc.)
- [ ] University Bachelors' degree in/related to coaching
- [ ] University Masters' degree in/related to coaching
- [ ] Doctorate in/related to coaching
- [ ] No formal qualification
- [ ] Other: ______________________

11. Are you currently in lock-down/have restricted movement due to the Covid-19 pandemic? (please chose one)

- ( ) Yes
- ( ) No
- ( ) I was never in lockdown/under restricted movement

12. How has the Covid-19 pandemic situation affected the athlete(s) you are responsible for in terms of daily training quality? (please chose one)

	1	2	3	4	5	
very negatively affected	○	○	○	○	○	very positively affected

13. How has the Covid-19 pandemic situation affected the athlete(s) you are responsible for in terms of relationships within the team (including supporting staff etc.)? (please chose one)

	1	2	3	4	5	
1 very negatively affected	○	○	○	○	○	very positively affected

14. How has the Covid-19 pandemic situation affected the athletes(s) you are responsible for in terms of maintaining/finding motivation to train? (please chose one)

	1	2	3	4	5	
1 very negatively affected	○	○	○	○	○	very positively affected

15. How concerned are you that your athletes have not maintained their training during the Covid-19 pandemic? (please chose one)

	1	2	3	4	5	
not concerned at all	○	○	○	○	○	very concerned

16. How do you think the restrictions caused by the Covid-19 pandemic affects/affected the skill development of your athletes? (please chose one)

	1	2	3	4	5	
very negatively	○	○	○	○	○	very positively

17. How do you perceive that your relationship with your athletes have been affected during the Covid-19 pandemic? (please chose one)

	1	2	3	4	5	
very negatively	☐	☐	☐	☐	☐	very positively

18. How do/did you communicate with your athletes before the Covid-19 pandemic? (tick all that applies)

- ☐ Email
- ☐ Online video (Skype,Zoometc.)
- ☐ Phone call
- ☐ SMS/WhatsApp/Messenger etc.
- ☐ In person
- ☐ Other: ________________

19. How do/did you communicate with your athletes during the Covid-19 pandemic? (tick all that applies)

- ☐ Email
- ☐ Online video (Skype,Zoom etc.)
- ☐ Phone call
- ☐ SMS/WhatsApp/Messenger etc.
- ☐ In person
- ☐ Other:

20. How often did you communicate with your athletes before the Covid-19pandemic? (tick all that applies)

- Multiple times per
- dayOnce per day
- Four to six times per week
- Onceper week
- Less than once per week

21. How often did you communicate with your athletes during the Covid-19 pandemic? (tick all that applies)

- Multiple times per
- dayOnce per day
- Four to six times per week
- Onceper week
- Less than once per week

22. Who is prescribing / prescribed the training program during the Covid-19pandemic? (please chose one)

- The athlete made up their own
- programMe, the coach
- Combination of athlete’s own training program and coach’s training
- programOther: ____________________

23. Which methods have you used to provide your athletes with training programmes/inspiration during the Covid-19 pandemic? (tick all that applies)

- Email them written programs
- Send links to online videos (already existing
-

online)Made my own videos

- [ ] Online collaboration tools (Google-docs, Teams, Slack
- [ ] etc.)Nothing
- [ ] Other: ______________________

24. How do/did you monitor your athletes' training during the Covid-19pandemic? (tick all that applies)

- [ ] Online training diary
- [ ] Smart technology (smart watches/Apps/GPS
- [ ] etc.)Email/phone call reports from athletes
- [ ] Other: ______________________

25. In your coaching role, who has been your main support/sparring partners during the Covid-19 pandemic? (tick all that applies)

- [ ] The athletes
- [ ] Partner/wife/husband/family
- [ ] (notcoach) Friend (not coach)
- [ ] Sporting
- [ ] federation(s)
- [ ] Other coaches

None

- [ ] Other: ______________________
