# Supplementary material for: New insights from Norwegian and Swedish sports coaches' employment, practices, and beliefs during the first COVID-19 restriction period
Source: Front Sports Act Living. 2023 Oct 27;5:1277228. doi: 10.3389/fspor.2023.1277228 (PMC10641774; doi:10.3389/fspor.2023.1277228)
Supplement: Supplementary file 3 [file Datasheet3.pdf]

Supplementary Table 1. Implications of the Covid-19 pandemic on employment situation and communication frequency with athletes.

	**Total**	**Norway**	**Sweden**
**How was your job position [as a coach] affected by the Covid-19 pandemic?**			
As usual	136 [39.1]	12 [8.5]	124 [59.9]
Home office	99 [28.5]	68 [48.2]	31 [15.0]
Furloughed	59 [17.0]	37 [26.2]	22 [10.6]
Lost job	7 [2.0]	1 [0.7]	6 [2.9]
Other	47 [13.5]	23 [16.3]	24 [11.6]
Increased workload	*6*	*0*	*6*
Reduced workload	*10*	*4*	*6*
No training	*12*	*12*	*0*
**How often did you communicate with athletes BEFORE the Covid-19 pandemic?**			
Several times/day	42 [12.1]	26 [18.4]	16 [7.7]
Once/day	30 [8.2]	19 [13.5]	11 [5.3]
4-6 times/week	99 [28.5]	37 [26.2]	62 [30.0]
2-3 times/week	87 [25.0]	35 [24.8]	52 [25.1]
Once/week	66 [19.0]	20 [14.2]	46 [22.2]
Less than once/week	24 [6.9]	4 [2.8]	20 [9.7]
**How often did you communicate with athletes DURING the Covid-19 pandemic?**			
Several times/day	11 [3.2]	7 [5.0]	4 [1.9]
Once/day	16 [4.6]	8 [5.7]	8 [3.9]
4-6 times/week	76 [21.8]	25 [17.7]	51 [24.6]
2-3 times/week	109 [31.3]	51 [36.2	58 [28.0]
Once/week	73 [21.0]	24 [17.0]	49 [23.7]
Less than once/week	63 [18.1]	26 [18.4]	37 [17.9]

Data is shown as numbers [percent within total sample or sub-group/country].

Supplementary Table 2. Subjective responses indicating the coaches' perceptions on the impacts of the Covid-19 pandemic.

	Total	Norway	Sweden
**Q1 - How do you think the restrictions caused by the Covid-19 pandemic affects/affected the skill development of your athletes?**			
Very negative	24 [6.9]	7 [5.0]	17 [8.2]
Somewhat negative	102 [29.3]	38 [27.0]	64 [30.9]
Neither negative nor positive	158 [45.4]	65 [46.1]	93 [44.9]
Somewhat positive	53 [15.2]	29 [29.6]	24 [11.6]
Very positive	11 [3.2]	2 [1.4]	9 [4.4]
**Q2 - How has the Covid-19 pandemic situation affected the athlete[s] you are responsible for in terms of relationships within the team [including supporting staff etc.]?**			
Very negative	29 [8.3]	10 [7.1]	19 [9.2]
Somewhat negative	99 [28.5]	39 [27.7]	60 [29.0]
Neither negative nor positive	166 [47.7]	71 [50.4]	95 [45.9]
Somewhat positive	43 [12.4]	19 [13.5]	24 [11.6]
Very positive	11 [3.2]	2 [1.4]	9 [4.4]
**Q3 - How has the Covid-19 pandemic situation affected the athletes[s] you are responsible for in terms of maintaining/finding motivation to train?**			
Very negative	30 [8.6]	11 [7.8]	19 [9.2]
Somewhat negative	136 [39.1]	50 [35.5]	86 [41.6]
Neither negative nor positive	101 [29.0]	47 [33.3]	54 [26.1]
Somewhat positive	61 [17.5]	27 [19.2]	34 [16.4]
Very positive	20 [5.8]	6 [4.3]	14 [6.8]
**Q4 - How has the Covid-19 pandemic situation affected the athlete[s] you are responsible for in terms of daily training quality?**			
Very negative	29 [8.3]	13 [9.2]	16 [7.7]
Somewhat negative	84 [24.1]	30 [21.3]	54 [26.1]
Neither negative nor positive	152 43.7]	60 [42.6]	92 [44.4]
Somewhat positive	69 [19.8]	36 [25.5]	33 [15.9]
Very positive	14 [4.0]	2 [1.4]	12 [5.8]
**Q5 - How do you perceive that your relationship with your athletes have been affected during the Covid-19 pandemic?**			
Very negative	7 [2.0]	4 [2.8]	3 [1.5]
Somewhat negative	50 [14.3]	16 [11.4]	34 [16.4]
Neither negative nor positive	199 [57.2]	79 [56.0]	120 [58.0]
Somewhat positive	78 [22.4]	36 [25.5]	42 [20.3]
Very positive	14 [4.0]	6 [4.3]	8 [3.9]

Data is shown as numbers [percent within total sample or sub-group/country].
